# Partial trisomy 2q33.3-q37.3 in a patient with an inverted duplicated neocentric marker chromosome

**DOI:** 10.1186/s13039-015-0111-1

**Published:** 2015-02-06

**Authors:** Ruiyu Ma, Ying Peng, Yanghui Zhang, Yan Xia, Guizhi Tang, Jiazhen Chang, Ruolan Guo, Baoheng Gui, Yanru Huang, Chen Chen, Desheng Liang, Lingqian Wu

**Affiliations:** State Key Laboratory of Medical Genetics, Central South University, 110 Xiangya Rd, Changsha, Hunan 410078 China; Department of Pediatrics, Xiangya Hospital, Central South University, Changsha, Hunan P.R China

**Keywords:** Growth retardation, Neocentromere, Partial trisomy 2q3, SNP array, sSMC

## Abstract

**Background:**

Increasing number of cases with small supernumerary marker chromosomes (sSMCs) without centromeric DNA and dozens of cases with trisomy 2q3 have been reported in recent years. However, cases of simultaneous sSMC and partial trisomy of chromosome 2q have been rarely described.

**Results:**

We report the case of a young girl patient with growth retardation and mild facial features due to a partial trisomy 2q33.3-37.3. The 34.3 Mb-duplication of the 2q33.3 to q37.3 region found in the patient constituted a supernumerary inverted duplicated neocentric marker chromosome.

**Conclusions:**

This is the first case of a patient with partial trisomy 2q33.3-37.3 presenting an inverted duplicated neocentric marker chromosome. Based on the case, this study will help further understanding the genotype/phenotype correlations of partial 2q3 duplication and exploring the relationship between neocentric sSMC and human diseases.

## Background

Small supernumerary marker chromosomes (sSMCs), which are detected in approximately 0.075% of prenatal specimens and 0.044% of newborn infants [1], are defined as structurally abnormal chromosomes that cannot be unambiguously identified or characterized by conventional banding cytogenetics alone [2]. In general, they are equal or smaller in size than chromosome 20 of the same metaphase spread. According to the literature, sSMCs are most often found in mentally retarded patients (0.288%), and followed by infertile patients (0.125%) [1]. sSMCs constitute a morphologically heterogeneous group appearing as different types of inverted duplicated-, ring-, or minute chromosomes. During meiosis and mitosis, the centromere controls the segregation of the genetic material. In the absence of endogenous centromeres, the rearranged chromosomes would either be lost or become acentric. While most human marker chromosomes appear to contain normal human centromeres with detectable alpha satellite DNA [3], stable sSMC that do not contain any known centromeric DNA have been increasingly reported in recent years. These sSMC contain a region called “neocentromere” acting as a functional centromere that has not been known to have a centromere function previously.

In this report, we present a case of partial trisomy 2q in a girl with neocentric sSMC. To the best of our knowledge, pure partial trisomy of chromosome 2q3 with the absence of associated monosomy is rare. This is the first report on the molecular cytogenetic characterization of a partial trisomy 2q33.3-37.3 with an inverted duplicated neocentric marker chromosome.

## Case presentation

### Case report

The patient, a 2-year-old girl, was referred to our center because of growth retardation, distinctive facial features, and chromosomal abnormalities identified at another hospital. She was delivered at term via cesarean section as the first child to non-consanguineous Chinese parents. Contributory family history was absent. Her weight at birth was 2.2 kg (~0.4th percentile), and no significant hypoxia, jaundice, or other complications were recorded at birth. The parents were 26 (mother) and 31 (father) years of age at the time of the birth. They were both healthy with normal karyotypes. Approximately 40 days into pregnancy, the mother had taken traditional Chinese medicine because of high temperature. After the baby was born, she had jaundice and was treated for six days. Although she could sit alone without assistance at nine months of age, she is still unable to walk alone at age 2, suggesting a delay in psychomotor milestones. Speech retardation also existed because she cannot have a dialogue with others except for some simple words such as “Mom” and “Grandma.” Physical examination showed a height of 86 cm (~50th percentile) and weight of 11 kg (~25th percentile). She had mild facial dysmorphic features, including ocular hypertelorism, epicanthus, upslanted palpebral fissures, flat nasal bridge, and protruding ear. No other obvious abnormalities were observed to date.

### Results

**Figure 1:**
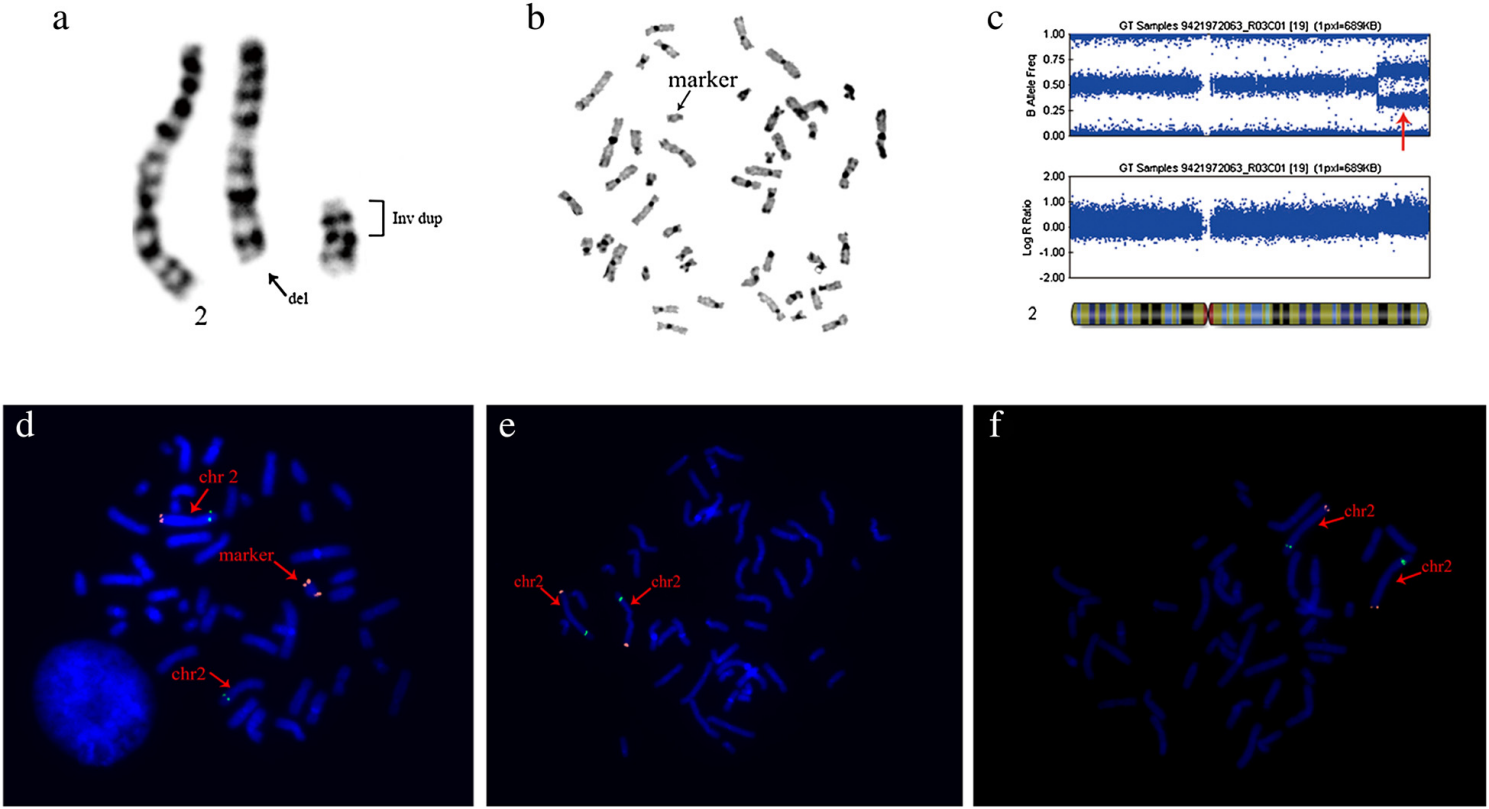
**Cytogenetic and molecular results. (a)** G-banding partial karyotypes of the patient, indicating a terminal deletion of 2q, accompanying with a marker chromosome. **(b)** C-banding showing no centromeric heterochromatin. **(c)** Copy number variation analysis for individual SNP loci along chromosome 2 using Illumina Human OmniZhongHua-8 Beadchip. B Allele Frequency and Log R show a gain of 34,268,291 bp (nt. 208775856–243044147) in terminal end of 2q (Red arrow ). **(d)** FISH results of the patient. FISH using RP11-526 L8 (orange) at 2q37.3 as a test probe and RP11-119B15 (green) at 2p22.3 as a control probe, showing an orange signal and a green signal on each of the two copies of only one chromosome 2, while the other one lacking the orange signal. Two orange hybridization signals for test probe on the SMC corresponding to an inverted duplication marker. **(e, f)** FISH results of the parents showing one hybridization signal for each probe on the two normal chromosomes 2.

Analysis of GTG-banded chromosomes in the proposita revealed a supernumerary marker in all analyzed metaphases; however, a 2q deletion was observed (Figure 1a). C-banding (Figure 1b) showed the absence of centromeric heterochromatin in the marker chromosome, suggesting the presence of a neocentric sSMC. The parents’ karyotypes were normal, indicating a de novo marker chromosome. SNP array analysis detected a 34.3 Mb-duplication (nt.208775856-243044147) of the 2q33.3 to q37.3 region (Figure 1c). The proximal breakpoint was mapped within a 7.5 kb region between SNPs rs2621470 and rs2621472 (nt.208768367-208775856) at 2q33.3, while the distal breakpoint was located in the last SNP rs12469535 at 2qter (position 243044147). Considering the result of banding cytogenetics and copy number variation analysis, we supposed that this supernumerary marker chromosome arose from chromosome 2. We confirmed the chromosome 2 origin with FISH analysis using a BAC clone RP11-526 L8 (orange) within the duplicated region in the patient and her parents (Figure 1d, e, and f). FISH studies also showed that the marker chromosome has some features of an isochromosome. The combined results proved that the supernumerary marker chromosome was derived de novo from chromosome 2 and corresponded to an inverted duplication conformation. Finally, we described the karyotype of the proposita as 47, XX, del(2)(pter → q33.3), +neo(2) (qter → q33.3 → neo → q33.3 → qter).

### Discussion

Till date, dozens of cases with trisomy 2q3 have been reported in the literature, most of which are associated with monosomy of another chromosome segment [4]. Majority are the result of an abnormal segregation of the parents chromosome rearrangement. In contrast, pure duplication of 2q3 is relatively rare. In the present study, we report a case of pure partial trisomy 2q33.3-q37.3. The proband, a 2-year-old girl displays mild facial dysmorphic features and has a delayed development similar to the known clinical features of the 2q3 duplication syndrome [5-7]. To our best knowledge, only 14 cases with isolated duplications of 2q overlapping 2q33-2qter have been previously described [4,8-19]. Majority of these cases (Table 1) are de novo and an inverted duplication is the most common form of rearrangement. Patients reported with duplication of 2q33-2qter also have similar clinical phenotypes, including variable developmental delays, facial and visceral anomalies, and trunk and limb malformations. Mild to moderate growth and mental retardation were documented in all cases except in the one without growth retardation [11]. Characteristic facial features were noticed in the neonatal period, including hypertelorism and epicanthic folds, broad flat nasal bridge, anteverted nostrils, long philtrum, thin upper lip, low-set ears, Cupid’s bow lip, and micrognathia. Minor visceral anomalies reported in some patients mainly involve cardiac, kidney or brain defects, and external genitalia malformations. In addition, common acral anomalies include fifth-finger clinodactyly, brachydactyly, and abnormal palmar or flexion creases. Overall, the clinical phenotypes of our proposita in this study are gentle and equivalent to those in the literature, yet visceral and limb malformations were not observed. Compared with other chromosome disease syndromes, most phenotypes of the 2q3 duplication syndrome are shared with other syndromes.

**Table 1:** **Reported cases with pure distal trisomy 2q overlapping 2q33-2qter**

**References**	**Duplication size**	**Type of rearrangement**	**Origin**	**Phenotype**		
				**Developmental delay**	**Facial/visceral anomalies**	**Trunk and limb malformations**
Dennis et al., [8]	2q33-2q37	Ins 12q23	Pat.	+	+/+	+
Yu and Chen, [9]	2q34-2q37	Inv dup	De novo	+	+/−	+
Kyllerman et al., [10] (2 cases)	2q34-2qter	Inv dup	Pat.	+	+/+	+
Dahoun-Hadorn and Bretton-Chappuis, [11]	2q35-2qter	Inv dup	De novo	+*	+/+	+
Romain et al., [12]	2q33.1-2q35	Dir dup	De novo	+	+/−	+
Fritz et al., [13]	2q35-2q37.1	Ins 17q25	De novo	+	+/+	+
Seidahmed et al., [14]	2q32-2q37	Inv dup	De novo	+	+/+	+
Angle et al., [15]	2q34-2qter	Inv dup	Mat.	+	+/+	+
Bonaglia et al., [16]	2q33-2q37	Inv dup	De novo	+	+/+	+
Bird and Mascarello, [17]	2q33.1-2q37.1	Dir dup	De novo	+	+/+	+
Slavotinek et al., [18]	2q33-2q37.3	Inv dup	De novo	+	+/−	+
Hermsen et al., [4]	2q35-q37.3	Trans dir dup	De novo	+	+/−	+
Pietrzak et al., [19]	2q35-q36	Sup r	De novo	+	+/−	+

Ins, insertion; Inv dup, inverted duplication; Dir dup, direct duplication; Trans dir dup, translocation direct duplication; Sup r, supernumerary ring; Mat., maternal; Pat., paternal.

*The mentally retarded child presented with comparable facial anomalies, hypospadias and bilateral cryptorchidism, but without growth retardation.

In this report, we describe the special rearrangement form in our patient, a de novo inverted duplication marker chromosome verified by cytogenetic and molecular analysis. Because the karyotype 47, XX, del(2)(pter → q33.3), +mar were detected in 100% cells and no heterochromatic region was found, we speculated that the marker carried “a newly derived centromere” to support its stability. The first human neocentromere lacking alpha satellite DNA was discovered by Voullaire in 1993 [20]. Since then, increasing numbers of new cases with neocentromere have been reported in the literature. Based on his analysis of 93 cases of neocentromere formation, Marshall et al. suggested “hotspots” of neocentromere formation. They found that certain regions of some chromosomes seemed particularly prone to form neocentromeres, such as 3q, 8p, 13q, and 15q, although the specific mechanism remains unclear [21]. Earlier studies have reported several similar cases with neocentromere markers that are originated from other chromosomes (Table 2). A review of these cases revealed that the major type of rearrangement of these mirror-image markers was terminal deletion accompanying with inverted duplication, which was also applicable to the present case. Case 6 was a little different from other cases for the presence of an isochromosome for the long arm of chromosome 16 and an acentric neocentric marker derived from the short arm of chromosome 16. Yet essentially, the likely mechanism of this complex rearrangement is also a deletion accompanying with inverted duplication. All the markers of these cases formed almost de novo. Mental retardation or delayed psychomotor development combined with physical handicaps could be observed in all probands of these cases, and the other clinical features of each proband were corresponding to the characteristics of each trisomy syndrome fundamentally. In our study, the neocentric sSMC is an inverted duplication chromosome derived from the distal region of chromosome 2q. To date, there are only two constitutional neocentromere cases with sSMC involving chromosome 2 described in the literature: one involving a ring marker chromosome from an interstitial deletion (paracentric) and the other a supernumerary ring. To the best of our knowledge, in partial trisomy 2q syndrome, this form of an inverted duplication neocentric chromosomal rearrangement with a deleted chromosome complementary for the region of duplication has not been previously reported.

**Table 2:** **Comparison of similar cases with inverted duplicated neocentric sSMC**

**Case number**	**Karyotype and grade of mosaicism**	**Type of rearrangement**	**Karyotype of parents**	**Reference on sSMC homepage** [11]
1	47,XX,del(1)(q32),+der(1)(qter → q32::q32 → qter)[100%]	Terminal deletion + Inv dup(possibly)	NOR	01-N-q32/1-1
2	47,XX,del(9)(p12),+der(9)(pter → p12::p12 → pter)[100%]	Terminal deletion + Inv dup	NOR	09-N-pt12/1-1
3	47,XY,del(11)(q22),+der(11)(qter → q22::q22 → qter)[100%]	Terminal deletion + Inv dup	NOR	11-N-qt22/1-1
4	47,XX,del(13)(q32.3),+der(13)(qter → q32.3::q32.3 → qter)[100%]	Terminal deletion + Inv dup	NA	13-N-qt32.3/2-1
5	47,XX,del(14)(q32.1),+der(14)(qter → q32.1::q32.1 → qter)[100%]	Terminal deletion + Inv dup	NOR	14-N-qt32.1/1-1
6	47,XY,i(16)(q10), +der(16)(p13.3p11.2)[100%]	Deletion + Iso	NOR	16-N-p11.2/1-1
7	47,XY,del(17)(q22q23),+der(17)(:q22 → q23::q23 → q22:)[100%]	Interstitial deletion + Inv dup	NOR	17-N-qt22/1-1
8	47,XX,del(20)(p11.2),+der(20)(pter → p11.2::p11.2 → pter)[100%]	Terminal deletion + Inv dup	NOR	20-N-pt11.2/1-1
9	47,XX,del(2)(q33.3),+der(2)(qter → q33.3::q33.3 → qter)[100%]	Terminal deletion + Inv dup	NOR	the present case

Inv dup, inverted duplication; Iso, isochromosomes; NA, not available for testing; NOR, normal.

**Figure 2:**
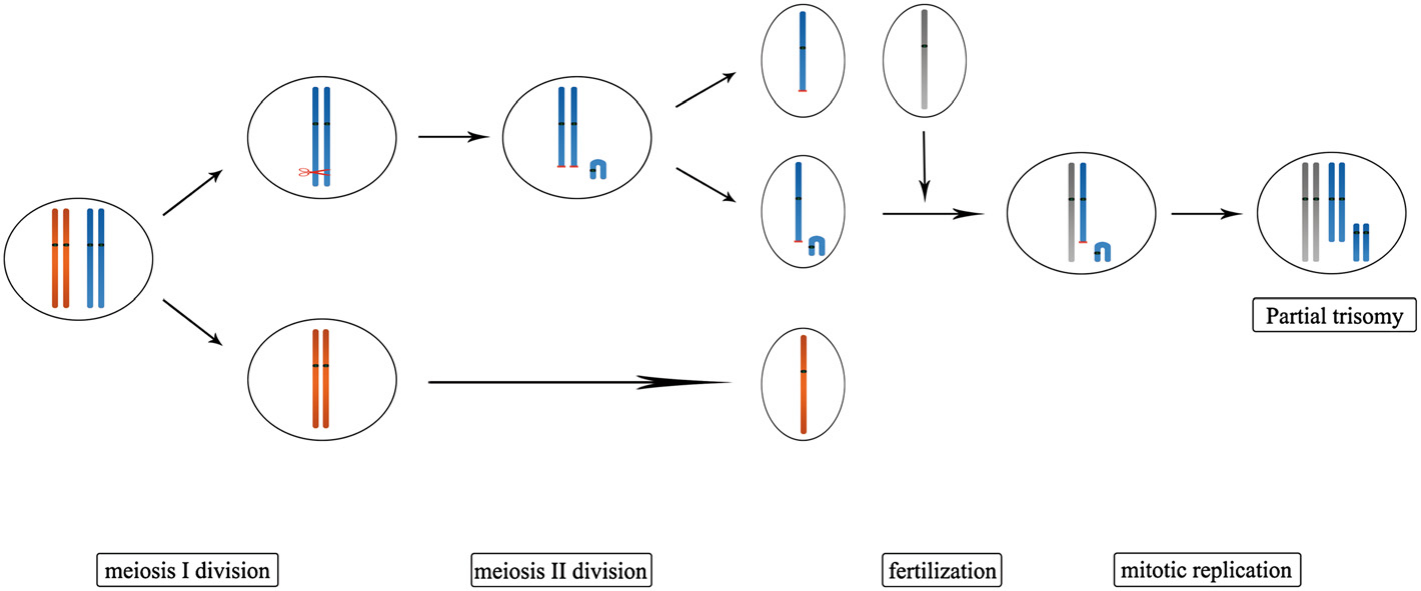
**Possible mechanisms for the formation of neocentric marker chromosome in the patient.** Chromatids breakage (red scissors) and a distal U-type exchange happened before sister chromatid separation during the meiosis II. Neocentromerization occurred once breakage happened. Deep color ovals are the centromeres, and the same one on the acentric fragment is neocentromere. The broken ends (red wave) of the centric fragment could be stabilized by telomere restitution. After zygogenesis and subsequent replication the result would be partial trisomy for the duplicated fragment.

**Figure 3:**
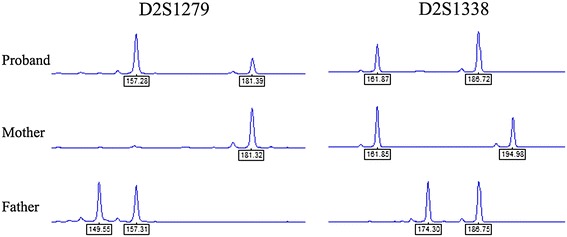
**Microsatellite analysis of markers D2S1279 and D2S1338 on ABI 3100 genetic analyser.** The 2:1 ratio between two fluorescent peaks areas in the proband indicated that she inherited a double dosage of one of the paternal alleles.

Considering the mechanism of neocentric sSMC formation, many different theories have been proposed. At present, ring and inv dup shaped neocentric sSMC are mainly reported. Ring neocentric chromosomes are mainly due to McClintock mechanism [22], which is in connection with a balanced situation in the carriers. For formation of inv dup shaped sSMC, as summarized by Liehr et al. [23], Marshall et al. [21] and Murmann et al. [24], these markers may form either in meiosis or mitosis. The majority are based on a U-type exchange between two homologous chromosomes at meiosis I, resulting in tetrasomy for the terminal chromosomal region present on the marker, while the remaining inv dup markers may come from other mechanisms leading to partial trisomy of that region in the genome. Our patient carries an apparent terminal deletion at chromosome 2q accompanied with a neocentric inverted duplication chromosome. Because no chimerism was found in our patient and in other patient reports with analogous rearrangements, we believed that the break could not occur during a mitotic event, otherwise mosaicism would be observed. In view of the above facts, we speculate that the most plausible explanation is a distal U-type exchange occurring between two chromatids of the same chromosome at meiosis II (Figure 2). A double-chromatid break occurred when the sister chromatids were about to separate during the second meiosis. Subsequently, two broken chromatids would be dragged into two hemizygotes in effect of traction, while the acentric chromosomal fragment resulting from crossover mistakes of the chromatids might segregate with one of them. Neocentromerization occurred at any location once the inv dup marker chromosome had been formed. After fertilization and subsequent replication in early zygote development, the endpoint would be partial trisomy in the form of the stable marker. This is the situation seen in our patient. To validate our speculation, we used simple quantitative fluorescent polymerase chain reaction (QF-PCR) to perform short tandem repeat analysis (Figure 3). Copy number variation occurring after the homologous chromosomes separation in meiosis can generate allele homologous repeats. Moreover, in trisomic diallelic cases, two out of three chromosomes share the same loci so that a pattern of 2:1 ratio between two peaks will be detected which coincides with our result. According to the evaluation criteria of QF-PCR [25], we can consider that the extra chromosome segment in our patient results from homologous paternal allele duplication. However, due to the lack of a satellite centromeric specific probe, the specific location of the neocentromere cannot be determined; whether it is an isochromosome is also unknown.

Among the reported cases, majority of neocentric marker chromosomes were derived from chromosomal arms or telomeric regions [21,26,27] containing a higher density of euchromatic sequences than centric sSMC. This may explain the strong correlation (~90 percentile) between neocentric sSMC and abnormal phenotype [28]. According to the literature, the critical region correlating with clinical features, including facial dysmorphism, postnatal growth retardation, syndactyly, and visceral hypoplasia, has been preliminarily mapped within the 2q34-qter interval [29]. We reviewed 56 virulence genes contained in the 2q33-2qter duplication region observed in our patient and found that four genes may determine the phenotype: *TM4SF20* (OMIM:615404), *PDE6D*(OMIM:602676), *HDAC4*(OMIM:605314), and *KIF1A*(OMIM:601255). Among patients with developmental delay, speech impairment, and/or brain imaging abnormalities, a heterozygous 4 kb-deletion of the *TM4SF20* was identified by Wiszniewski et al. [30]. Thomas et al. reported a *PDE6D* mutation in two siblings with Joubert syndrome displaying intrauterine growth retardation, facial dysmorphism, postaxial polydactyly of the feet, syndactyly, and other symptoms [31]. Patients with brachydactyly mental retardation syndrome resulting from a mutation, deletion, or interruption in *HDAC4* have been described with an analogous phenotype in a report [32]. Mutations in *KIF1A* caused nonsyndromic intellectual disability in a patient described by Hamdan et al. [33]. The phenotype associated with these four disease-causing genes are overlapping with the one observed in our patient, although no syndactyly and visceral anomalies were seen in our patient. This discrepancy may be due to the fact that the pathogenicity of genes is mainly determined by interrupted or deleted variants rather than duplications. Genes are dosage-sensitive and become defective by loss rather than by gain of function. Furthermore, we also noticed that the chromosome 2q35 duplication syndrome (OMIM:185900) was located within the duplication interval in our patient. This syndrome is characterized by syndactyly type I and Philadelphia-type craniosynostosis. Klopocki et al. [34] identified a 59 kb microduplication at the *IHH* (OMIM:600726) locus on chromosome 2q35 and a minimum region of 9.1 kb region located 40 kb 5’ of the *IHH* gene, in three families associated with variable degrees of syndactyly and craniosynostosis. Our patient carries this microduplication but does not have these characteristics, showing only mild facial dysmorphism and delayed psychomotor development. One reason may be because of the broader duplicated region which encompasses more genes and gene regulatory regions. A large fragment repeat of the gene regulatory region may act as a group affecting gene expression differently, by controlling the expression of genes either within the region or outside, therefore, resulting in different phenotypes. Moreover, this novel rearrangement may lead to chromatin changes, and the presence of the neocentromere in the marker chromosome may influence gene expression [35]. Finally, it is difficult to explain a clear genotype/phenotype correlation for the 2q3 duplication syndrome because of variable clinical situations and the ambiguous breakpoints. The specific pathogenesis remains to be explored with precise breakpoint position mapping and related functional studies.

## Conclusions

In conclusion, we report the first case of a patient with partial trisomy 2q33.3-37.3 presenting an inverted duplicated neocentric marker chromosome. Our patient had only facial dysmorphism and developmental delay as opposed to the previously described phenotype found with pure partial trisomy of chromosome 2q3. We defined the origin of the duplication fragment and analyzed the rearrangement and the patient’s phenotype which will help further understanding of the genotype/phenotype correlations of partial 2q3 duplication and exploring the relationship between neocentric sSMC and human diseases.

### Methods

Cytogenetic analyses were performed on metaphase chromosomes of the patient and her parents derived from peripheral blood lymphocytes cultures. G-banding (400–550 bands) was performed first, according to the standard procedure. However, to further determine whether sSMCs found in GTG-banding karyotype contained centromeres, we performed C-banding which highlighted heterochromatic regions.

Genomic DNA of the patient was extracted from fresh peripheral blood using the standard phenol/chloroform method. SNP array analysis of the extracted DNA was performed using the Human OmniZhongHua-8 Beadchip (Illumina, San Diego, CA) with an average resolution of 3.34 Kb. The arrays were scanned on a microarray scanner and analyzed using GenomeStudio (cnv Partition Plug-in v3.1.6) software. The operative procedures mentioned above were all performed according to the manufacturer’s recommended protocols (www.illumina.com).

In order to verify the possible pathogenic copy number variations and determine their origin, FISH analysis was performed on metaphase or interphase chromosomes from the patient and her parents. We used two BAC clone probes, RP11-526 L8 (orange) at 2q37.3 as a test probe, and RP11-119B15 (green) at 2p22.3 as a control probe.

## Consent

Written informed consent was obtained from the parents of the patient for publication of this Case report. A copy of the written consent is available for review by the Editor-in-Chief of this journal.

## Abbreviations

sSMCs: Small supernumerary marker chromosomes
SNP array: Single nucleotide polymorphism array
FISH: Fluorescent In Situ Hybridization
BAC: Bacterial artificial chromosome
QF-PCR: Quantitative fluorescent polymerase chain reaction
